# Medical Simulation: The Least Advertised and Most Versatile Weapon in Pandemic

**DOI:** 10.3389/fmed.2020.582150

**Published:** 2020-10-09

**Authors:** Valentin Favier, Sam J. Daniel, Marc Braun, Patrice Gallet

**Affiliations:** ^1^Department of Otolaryngology-Head and Neck Surgery, Gui de Chauliac Hospital, University Hospital of Montpellier, Montpellier, France; ^2^Aide à la Décision Médicale Personnalisée, Montpellier University, Montpellier, France; ^3^Department of Otolaryngology-Head and Neck Surgery, McGill University, Montreal, QC, Canada; ^4^Virtual Hospital of Lorraine, University of Lorraine, Nancy, France

**Keywords:** pandemic, COVID-19, *in situ* simulation, simulation center, adaptability

## Introduction

From America to Europe, world leaders declared war on coronavirus disease (COVID-19), an invisible, poorly understood enemy. Most countries and health-care providers were baffled by the rapid pace of the pandemic. The World Health Organization (WHO) declared the outbreak as a public health emergency of international concern on January 30th. Through simulation scenarios published the next day, it highlighted the need for being prepared and organized to march off into battle. Initial research focused on understanding the virus, testing drugs, and developing strategies. In this fight against the pandemic, a “new” medical weapon has emerged: medical simulation.

Simulation is an agile, concrete, and mobile multi-tool (1), useful for learning in all domains (knowledge, skills, and behavior) (2). It may be used to safely train professionals in real-like conditions (3) at several levels. In times of crisis, simulation is an ideal medium to update and enhance competencies and adapt practices, particularly constantly evolving practices. At a team level, simulation helps to face complex clinical situations like cardiac arrest in an infected patient (4) or prone positioning for managing respiratory distress. The ability of simulation to promote teamwork is also decisive (5). At a department level, a simulation may optimize the patients and working flow within new constraints. At a personal level, simulation equips one with behaviors and skills for safely donning and doffing, as well as technical skills such as intubation with minimal aerosol exposure.

Therefore, simulation in a COVID-19 context is akin to a “Swiss Army knife,” as it carries with it extreme utility and applies to several scenarios at hand. This weapon is loaded in simulation centers and its use adapted on sites (*in situ*). On the basis of the experience of two universities located in heavily affected areas (University of Lorraine, Nancy, France, and McGill University, Montréal, Canada), we describe here how this “Swiss Army knife” helped in adapting the answer to COVID-19 with two preferred complementary approaches: *ex situ* and *in situ* simulations, respectively.

## Roles of Simulation in Health Care to Face the Pandemic

Our simulation centers were leaders in the battle against COVID-19. In March 2020, in Nancy, the Le Centre Universitaire d'Enseignement par la Simulation—l'Hôpital Virtuel de Lorraine opened continuously for 1 month: 350 health professionals, from private and public hospitals, received *ex situ* training by simulation.Simulation sessions were designed to actualize knowledge and retrain and adapt procedures, using COVID-19 personal protective equipment. Thus, simulation centers offered an ideal framework to learn how to adapt aerosol-generating procedures to face COVID-19 and helped in disseminating related learning society recommendations. Despite a stressful context, it enabled to train people in a secure and reassuring environment. Older colleagues and retirees mobilized for backup were transmitting their expertise without being exposed to the battlefield and preparing care workers to adapt to a new work reality and to acquire competencies outside their habitual scope of practice (e.g., a surgeon performing the duty of an intensive care nurse). The simulation center acts as an accelerator for new skills acquisition and is an integral part of continuing education programs that maintain and reinforce these skills. As an example, we developed a course to train ear, nose, and throat (ENT) surgeons to perform percutaneous dilatational tracheostomy in intensive care units (6), as recommended by the French ENT Society (Société Française d'Oto-Rhino-Laryngologie). The course gathered ENT surgeons from both public and military hospitals. A brief introduction of the technical aspect of the procedure was followed by training on a low-cost homemade low-fidelity simulator, then various simulation scenarios (common procedure, complicated procedure, and decision making) in a high-fidelity environment. Donning and doffing techniques for COVID-19 were also part of the course. We also tested innovative protective equipment to limit aerosolization during procedures. Thanks to these courses, we were able to propose a checklist to prepare fully before entering the intensive care unit room (6).

At a larger scale, simulation centers were also placed at the front of research and development against COVID-19. This marked a historic turning point for simulation practice and might drive its evolution soon. Particular attention was paid to the protection of caregivers (7), with the development and evaluation of personal protective equipment (8). Studies reported the use of fluorescence to simulate and visualize droplets generated during airway management procedures, providing a direct visualization under ultraviolet light (9). Simulation centers, which ordinarily bring engineers from various backgrounds and health-care professionals together, have played this role even more widely (10). They acted as catalysts for ideas.

The simulation also helped craft new equipment and train teams that faced a critical shortage of supplies. Many solutions were developed, such as modified full-face snorkel masks for non-invasive ventilation (11), adapted ventilators to allow ventilation of multiple simultaneous patients (12), and techniques to help teams move intensive care unit patients to spare human resources, like the use of exoskeletons to help intensive care units with prone positioning (13). Thanks to simulation centers, these innovations could be designed, tested, and adapted in real-like conditions and in a record time (14). During crises, simulation centers may act as the rear base of the fight, becoming training camps for care teams and a place for developing strategies.

However, medical simulation is not restricted to simulation centers. In the short history of the COVID-19 pandemic, the first reported feat of arms of *in situ* simulation (15) tested the preparedness of teams and isolation of operating rooms in Singapore. Bringing simulation to the hospital grounds made possible large-scale training, facilitated by the proximity of real equipment to full teams (16). *In situ* simulation may help to improve plans by discovering their practical shortcomings and allows operational feedback such as lack of medical devices or human resources. At the McGill University Health Center, a number of *in situ* simulations were developed, as illustrated in Table 1. These improved team efficiency (17) and patient safety and became models for other hospitals, as many were posted freely on social media platforms and forums (WhatsApp “covid” group). *In situ* simulation also enables the extension and repetition of the training outside of the usually formatted curriculum. With teams and equipment on-site, it becomes possible to carry out simulations and training on a large scale (18). It also helps the organization adapt quickly by enabling teams to continuously update actions and plans against rapidly shifting challenges (19). Examples of simulation scenarios applied *in situ* at McGill are freely available online (20).

**Table 1:** *In situ* simulation scenarios during COVID-19 outbreak.

**Team protection**
Donning and doffing: surgeons, nurses, respiratory therapist, anesthesiology, emergency room, intensive care unit, residents, medical students, allied health professionals
Cardiopulmonary resuscitation in a coronavirus disease-positive patient
N-95 fit check, N-95 application and removal, and surgical scrubs removal
**Minimizing aerosol generation/protecting airway**
Rigid bronchoscopy, suspension laryngoscopy, tracheostomy, and sinus surgery
Intubation, extubation, difficult airway, endoscopies, and resuscitation
Transport throughout the hospital: operating room/emergency room/elevators/intensive care unit
**Clinic space reorganization**
Performing endoscopies in negative pressure rooms
Social distancing measures, scheduling, and patient flow
**Clinical care reorganization**
Telemedicine scenarios, advance health care directive, and levels of care
Dealing with clinical uncertainty
Prioritization of clinical consultations/operating room case waitlist

## Discussion

*In situ* simulation may encounter major limitations in hospitals: for instance, care units are not optimized for audiovisual assessment, and clinical activity imposes its own constraints (efficiency, the pressure to succeed, workforce issues, etc.) to the detriment of medical simulation. Fortunately, medical simulation is increasingly entering into hospitals in all its aspects: trainees are committed to the field with their trainers at their side. Hence, the influence of medical simulation is changing the culture of the physicians, who are increasingly applying the fundamentals of simulation in hospitals: systematic feedback, briefings, and debriefings. Some institutions took this to heart and integrated simulation centers directly on-site, like the Shriner's Simulation Center in Montréal or a branch of the Virtual Hospital of Lorraine in the University Hospital of Nancy. Easy access to simulation centers is critical to facilitate the coming of hospital staff and equipment. In the fight against COVID-19, simulation centers can repurpose simulation equipment for clinical use (21). The simulation center of Nancy directly engaged its training equipment on the combat front by providing the hospital with several supplies, including ventilators, protective equipment, syringe pumps, and video laryngoscopes.

Thus, in addition to its usefulness for learning, medical simulation proved to be effective in diagnosing shortcomings and establishing strategies. What was true during the outbreak certainly applies in all circumstances. The simulation creates more alert workers, trains reflective practitioners, and makes them aware of team working difficulties. Applied in everyday life, a medical simulation may help in reducing risks and increasing the quality and safety of care. Simulation may help in building teams and creating emulation to find solutions to potential problems and thereby instills a culture and a pattern of cognition among health-care workers and simulation trainers. Although trying to define medical simulation's fields of application is already restricting its use: by definition, a Swiss Army knife can be useful for unintended uses. Therefore, the investment in simulation is definitely a good placement for the future.

However, investing in a Swiss Army knife is only useful if it does not stay in your pocket, and the investment must be made in a simulation capable of being deployed in the field. The future of health-care simulation will either be agile or it would not be. This implies thinking upstream about resource allocation and scriptwriting. Future investments should be designed with this idea of mobile deployment in mind. Of course, simulation centers must be able to (1) host planned simulation training courses, but they should also enable to (2) create mobile teams for *in situ* simulation, (3) contribute to the war effort by supplying devices and expert advice in the field, (4) identify the changing needs of care workers and teams, (5) continuously adapt training to the clinical situations encountered, and (6) draw lessons from health-care crisis to upgrade daily practice.

The existence of prior links between simulation centers (or simulation teams) and institutions is a key to the successful use of simulation (in centers or *in situ*) in times of crisis. The simulation needs directly came from the field: simulation trainers were at the bedside, facing practical shortcomings. Trainers summarized team needs, then created and adapted simulation solutions. However, this was possible only because of the preexisting culture of simulation and the well-established partnership between our hospitals and simulation centers.

In the case of COVID-19, perhaps, our most potent weapon is this Swiss Army knife model, vital to our capacity to adapt swiftly. This weapon developed quickly and effectively through seamless partnerships between our university simulation centers and the hospitals with their *in situ* teams transcending the current crisis and enhancing our ability and our nimbleness in fighting this war. National and international simulation networks have contributed to the fight against COVID-19, especially for the dissemination of simulation scenarios and courses to help the simulation community. In Canada, Simulation Canada had quickly proposed dedicated online courses to face the pandemic (22) and shared simulation scenarios to help the simulation community, which was very helpful. The WHO made a great effort by providing COVID-19 tabletop exercise packages (23) to prepare countries for the outbreak. In July 2020, they also published a technical guidance (24) preconizing that “countries should be actively engaging all relevant ministries and stakeholders across multiple sectors (…) so as to broaden health security capacity building, including through simulation exercises during opportune periods.”

However, the COVID-19 outbreak has also unmasked disparities in access to simulation and a lack of organization. In France, for instance, the collaboration between simulation centers and most of the main private or university hospitals is well-established, but the vast majority of health-care facilities are still not part of a simulation network. Similarly, in other countries, access to simulation is not systematic even for university hospitals: in an international survey on simulation among ENT surgeons (residents and faculty staff) during the pandemic, more than 20% stated they did not have access to simulation resources in their institution (whatever the type of simulation), mainly in South America and in Europe (25). Furthermore, some countries had to close their simulation centers during the crisis (26), which has restrained access to simulation resources.

The current way of doing (building emergency responses based on requests in the field) has allowed a great adaptation to the needs but also turned out to be highly improvised and disparate. A well-thought plan is half the battle, and the COVID-19 outbreak clearly exposes the need for a much more organized, planned response. To illustrate the importance of an established plan, high-speed trains were used in France to transport critically ill COVID-19 patients from Nancy to other regions where hospitals had more capacity. This exceptional deployment was not conceived in an emergency but simulated a few years ago as a potential response to terrorist attacks. By simulating future crises, unlikely or never encountered situations, lessons can be learned for tomorrow, and plans can be developed. Coordination and allocation of resources on a large scale in times of crisis are also a major challenge, which could benefit from a dedicated steering committee that goes beyond local networks. Such committees could benefit policymakers, head of simulation centers, and care workers (nurses and physicians at least) as suggested by the WHO (24). A collective thinking process will indubitably improve our flexibility to face future problems and help answer the questions raised by this vision, i.e., to choose (i) leadership, (ii) steering indicators, (iii) means to collect needs and relevant information, and (iv) means to monitor and evaluate outcomes.

Other health crises will likely occur in the coming years. Caregiver training is paramount, and simulation is an attractive option to achieve this. Access to health-care simulation must be optimized, and a collective reflection must be carried out to this end: simulation definitely has its place in the centers, but pre-established partnerships and an army of trainers are the keys to rapid *in situ* deployment. Simulation is a “Swiss Army knife” that must be in everyone's pocket.

## Author Contributions

VF, PG, SD, and MB: design. VF and PG: first draft of the manuscript. SD: supervision. SD and MB: critical revision. All authors contributed to the article and approved the submitted version.

### Conflict of Interest

The authors declare that the research was conducted in the absence of any commercial or financial relationships that could be construed as a potential conflict of interest.
